# Separation of Radionuclides from Spent Decontamination Fluids via Adsorption onto Titanium Dioxide Nanotubes after Photocatalytic Degradation

**DOI:** 10.3390/nano10081553

**Published:** 2020-08-07

**Authors:** Monika Lyczko, Barbara Wiaderek, Aleksander Bilewicz

**Affiliations:** Institute of Nuclear Chemistry and Technology, 03-195 Warsaw, Poland; b.wiaderek86@gmail.com (B.W.); abilewic@ichtj.waw.pl (A.B.)

**Keywords:** titanium dioxide, titanium nanotubes, decontamination fluids, photocatalytic degradation

## Abstract

A one-step process combining the photocatalytic degradation of radionuclide complexes and the adsorption of liberated radionuclides on titanium dioxide nanotubes was developed and tested for the purification of aqueous waste produced from chemical decontamination of nuclear power plant circuit components. Among the tested forms of TiO_2_, only nanotubes exhibit both high photocatalytic activity and sorption ability, which support their application in a one-step purification process. The obtained results indicate that the photocatalytic degradation of complexes followed by the sorption of the radionuclides onto TiO_2_ nanotubes offers a promising route for treating spent decontamination fluids.

## 1. Introduction

During the operation of a nuclear power plant (NPP), high temperature water is continuously recirculated through the reactor coolant system. The operational parameters often lead to corrosion on the metal surface and erosion of the corrosion film. The metallic particles and metal cations that are released from the solid surface into the coolant can be deposited on the piping and/or the reactor core surfaces, where they are exposed to neutrons. Following activation, the material is either dissolved or eroded from the surface, such that it is transported by the reactor coolant to a different area of the reactor coolant system. Among the radioactive corrosion products, the most important are long-lived nuclides (e.g., ^60^Co, ^65^Zn, ^110m^Ag, and ^54^Mn) and short-lived nuclides (e.g., ^58^Co, ^59^Fe, ^51^Cr, and ^124^Sb). However, the biggest problem is posed by long-lived ^60^Co, generated from ^59^Co, which is the natural cobalt isotope present in the construction materials. The ^60^Co is produced when ^59^Co is activated by thermal neutrons in an (n, γ) reaction [1]. Irradiated corrosion products increase the doses received by personnel in the nuclear power plant, and they should be removed from these elements in order to adhere to the guiding principle of radiation safety, “ALARA” (as low as reasonably achievable).

The most effective and common decontamination process employs chemical methods. The basic solutions used for decontamination are liquids containing oxalic and citric acid (e.g., oxalates and citrates), and sometimes other chelating compounds, such as ethylenediaminetetraacetic acid (EDTA) to bind metal cations. In many decontamination procedures, peroxidation is used in the first stage of purification to facilitate sediment dissolution. 

Chemical decontamination processes generate tons of fluid waste containing chelators, oxidants, salts, and chelated radioactive corrosion products, which accumulate and concentrate in evaporator concentrates. Various separation methods are used to remove radionuclides from decontamination concentrates, including co-precipitation, ion exchange, and/or adsorption on inorganic materials [2]. Currently, sorption processes are the most common for isolating radionuclides from liquid radioactive waste, and they are even efficient for very salty solutions containing high concentrations of competing cations. Unfortunately, the presence of chemical substances that form complexes with the radionuclides drastically decreases sorption efficiency and makes it difficult to separate the radionuclides from decontamination solutions [3]. In addition, the presence of radionuclide complexes in solid radioactive wastes may result in elevated leachability and higher mobility of the radionuclide contaminants. In most cases, the long-lived radionuclides in decontamination fluids exist in complexed forms. The exceptions are cesium radionuclides (e.g., ^134^Cs, ^135^Cs, and ^137^Cs), which do not form stable complexes as alkali metal cations, but rather occur simply in the form of hydrated cations. The ^65^Zn and ^60^Co radionuclides exist as complexes in decontamination fluids, hence they each demonstrate relatively lower sorption from those solutions. However, the separation of these radionuclides is particularly important because of their long half-lives and emission of high energy gamma radiation.

In fact, one of the most interesting sorbents for this purpose is titanium dioxide (TiO_2_), which is a well-known inorganic ion exchanger that exhibits high affinity for transition metal cations, such as Co^2+^, Zn^2+^, Mn^2+^, and Ag^+^ [4,5]. In addition to its favorable adsorption properties, TiO_2_ also has strong photocatalytic properties [6].

TiO_2_ exists in amorphous and crystalline rutile, anatase, and brookite forms. Generally, the rutile phase is the most stable, while the other two phases are metastable [7]. These forms possess different structural and physical properties, which influence their sorption properties and photocatalytic activity. The most photoactive form of TiO_2_ is anatase, which is characterized by a high degree of surface hydroxylation, a large specific surface area, and a band gap energy of *E*g = 3.23 eV (384 nm). Rutile usually demonstrates much lower efficiency in photocatalytic processes, despite its smaller *E*g = 3.02 eV (411 nm). This is mainly a result of the difference in the recombination rates of electron-hole pairs [8]. The amorphous form of TiO_2_ is considered to be essentially photocatalytically inactive [9]. 

The aim of this work is to combine the sorption and photocatalytic properties of TiO_2_ nanotubes for the separation of the two most dangerous radionuclides (^60^Co and ^65^Zn) from decontamination fluids. In previous work from our group, we determined that TiO_2_ in the form of nanotubes has a high ion exchange capacity (>1 mmol/g) and a very large specific surface area [10]. Considering their high photocatalytic properties, we propose a simple one-step process, in which photocatalytic decomposition of the ^65^Zn and ^60^Co complexes and adsorption of the released radionuclides takes place on TiO_2_ nanotubes [11]. 

## 2. Materials and Methods 

### 2.1. Materials 

The reagents were purchased from Sigma-Aldrich (Saint Louis, MO, USA): titanium dioxide nanopowder ~21 nm, 99.5% trace metal basis (P25), titanium dioxide powder (anatase), and 99.8% metal basis; or from POCH S.A.: titanium(IV) chloride, sodium hydroxide microgranules (NaOH), potassium hydroxide (KOH), nitric acid min. 65% (HNO_3_), hydrochloric acid 35–38% (HCl), sodium citrate (Na_3_C_6_H_5_O_7_), ethylenediaminetetraacetic acid (EDTA), citric acid (C_6_H_8_O_7_), oxalic acid (C_2_H_2_O_4_), ammonia 25% (NH_3_), orthophosphoric acid 75%, and titrafix (TM). All of the reagents were of analytical quality and used without pre-treatment. ^60^Co, ^65^Zn, and ^85^Sr radioisotopes were received as solutions of 0.1 HCl (1 mCi) from NCBJ-POLATOM Isotope Center in Świerk (Świerk, Poland).

The synthesis of TiO_2_ nanotubes was carried out by the hydrothermal method as described by Kasuga et al. [12]. Briefly, 1.5–1.7 g of TiO_2_-anatase precursor was mixed with 70 mL of 10 M NaOH and the suspension was placed into a Teflon (polytetrafluoroethylene, PTFE)-lined autoclave and heated at 140 °C for 72 h with constant stirring. After cooling to room temperature, the obtained product was filtered, rinsed with water, rinsed next with 0.1 M HCl, and rinsed again several times with water until the pH of the supernatant solution reached a constant value of ca. 8–9.

The synthesis of amorphous TiO_2_ was carried out as follows: a precipitate of titanium dioxide was obtained from the titanium tetrachloride solution. TiCl_4_ was first diluted with water to a concentration of 0.1 M. Then, 0.1 M NaOH was added in small portions to a 0.1 M solution of titanium tetrachloride until the pH reached about 10. The obtained precipitate was left for 24 h, then washed with distilled water. The obtained gel was dried at 40 °C for 24 h. The resulting glassy grains were crushed with the addition of water and dried again for 12 h at 30 °C. After grinding, the obtained grains had a diameter of 0.1–0.4 mm. The appropriate forms were obtained after filtration of the obtained powder on a glass filtration set with a tread membrane (pore diameter: 0.8 µm).

### 2.2. Measurements

The morphology of the titanate nanotubes was confirmed by a scanning electron microscope (SEM) (carried out on ZEISS “ULTRA plus” Ultra-High-Resolution Imaging with microanalysis system EDS Bruker Quantax 400, Institute of High pressure Physics, Polish Academy of Sciences, Warsaw), and a transmission electron microscope (TEM) (carried out on Zeiss LEO 912AB, Warsaw University, Warsaw, Poland). 

Radioactivity measurements were carried out using a HPGe detector of gamma radiation connected to the multichannel analyzer Canberra Packard (Oak Ridge, TN, USA) with software Genie 2000 (version 3.2, Canberra Packard, Oak Ridge, TN, USA) detection range 10–5000 keV with a resolution of 0.8 at 5.9 keV, 1.0 at 123 keV, and 1.9 at 1332 keV. For some γ measurements, the automatic gamma counter Wizard 2480 (Perkin Elmer, Downers Grove, IL, USA) with NaI (Tl) detector, detection range 15–2000 keV, was also applied.

### 2.3. Adsorption of Radionuclides on Sorbents from Solutions of Simulated Decontamination Fluids

The sorption of radionuclide ions was performed on amorphous titanium (IV) oxide, commercially available P25 spherical nanoparticles, and TiO_2_ nanotubes in different pH, appropriate concentration of NaNO_3_ or KNO_3_, or appropriate concentration of complexing ligands (citric acid, oxalic acid, EDTA).

For example, 10 mg of the sorbent was added to 10 mL of a solution of 10^−3^ to 10^−1^ M citric and oxalic acid and 10^−5^ to 10^−2^ M EDTA containing ^60^Co^2+^ and ^65^Zn^2+^ radionuclides. The samples were mixed on a circular mixer. After 20 min of mixing, 1 mL of the suspension was taken into an Eppendorf tube, then after centrifugation, 0.5 mL of the supernatant solution was collected for radioactivity measurement.

The distribution coefficient (*K_d_*) values were calculated according to the following equation:

(1) $$K_{d}=\frac{\left(A_{i}-A_{eq}\right)}{A_{eq}}\;\frac{V}{m}$$

where *A_i_* and *A_eq_*denote the radioactivity of the initial solution and at the equilibrium, respectively, *V* is the volume (mL) of the solution, and *m* (g) is the mass of the titanium oxide adsorbent.

### 2.4. Photocatalytic Degradation 

**Figure 1:**
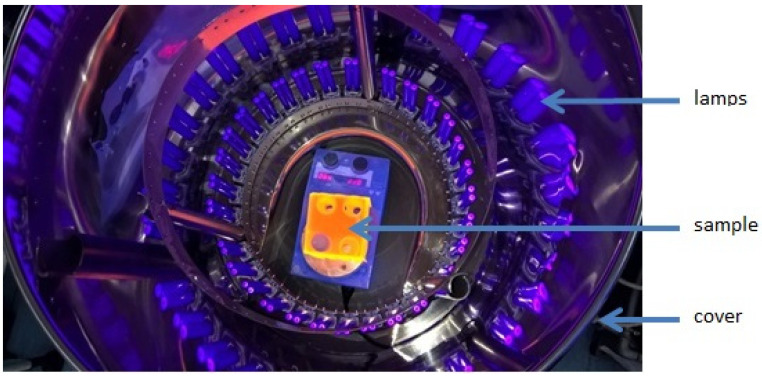
The interior of a photoreactor constructed by our team.

Photocatalytic degradation of the components of simulated decontamination fluids was carried out in the homemade photoreactor as presented in Figure 1.

A glass quartz beaker containing the tested TiO_2_ photocatalyst (concentration from 0.025 g/L to 0.25 g/L) and 20 mL of a solution of simulated decontamination fluids containing ^60^Co^2+^ and ^65^Zn^2+^ complexes was placed in the photoreactor for 2 min. The initial concentrations of the complexing agents in the solution of simulated decontamination fluids were: for EDTA 5 × 10^−4^ M, for oxalic acid 10^−3^ M, and for citric acid 10^−3^ M; pH = 4. The solution also contained 0.1 M Na^+^ cations. The power of UV radiation sources was 500 W, and the wavelength was 180–400 nm.

## 3. Results and Discussion

### 3.1. Sorption of ^60^Co and ^65^Zn on TiO_2_ Nanotubes 

The solution of spent decontamination products generated during the decontamination of cooling circuits, steam generators, and circulating pumps at NPPs may contain (i) complexing agents such as citric and oxalic acids or EDTA, (ii) oxidizing agents such as KMnO_4_, and (iii) radionuclidic contaminants such as ^60^Co, ^65^Zn, ^110m^Ag, ^58^Co, ^124^Sb, ^59^Fe, or ^54^Mn. It is generally beneficial to separate the radionuclides from the decontamination solution and immobilize them in permanent matrices in order to reduce the amount of overall waste. Among the various established methods for removal of radionuclides from liquid radioactive wastes that also contain complexation agents, photo-oxidation via UV irradiation followed by adsorption provides the best solution. In fact, TiO_2_ is the most efficient material for supporting the photodegradation of organic materials with UV radiation. On the other hand, it is one of the most effective inorganic ion exchangers for adsorption of metal cations. The ideal approach to separate radionuclides from decontamination solutions would therefore involve a one-step integrated process wherein the TiO_2_ would serve both as a photocatalyst and as a sorbent for the radionuclides liberated from the complexes. Unfortunately, Sebesta et al. [13] demonstrated that materials embodying favorable photocatalytic properties that also have high specific surface areas, such as Degussa P25 TiO_2_ and pure rutile TiO_2_, are weak metal sorbents. In contrast, amorphous TiO_2_ is an efficient metal cation sorbent [4]; however, it does not exhibit photocatalytic properties. Therefore, the authors concluded that a one-step process is difficult to implement, and the most promising route for treating these types of wastes is instead a two-step process consisting of photocatalytic degradation of citrate, oxalate, and EDTA complexes on photoactive TiO_2_ grains with subsequent adsorption of the liberated radionuclides onto a strongly acidic ion exchanger [13]. 

**Figure 2:**
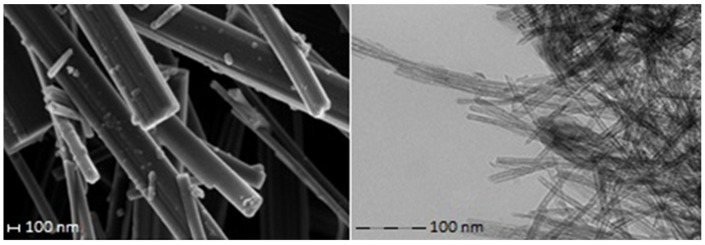
SEM (left) and TEM (right) images of synthesized nanotubes.

Recently synthesized TiO_2_ nanoforms, such as nanotubes (Figure 2), nanowires, nanoribbons, and nanofibers, have significantly expanded the range of TiO_2_ forms exhibiting photocatalytic properties. After carrying out adsorption experiments, we found that TiO_2_ nanotubes were also effective cation sorbents [10]. Considering these properties of TiO_2_ nanotubes, we propose that they can be applied for single-step isolation of radionuclides from decontamination fluids. 

In this work, we chose ^65^Zn and ^60^Co as two representative radionuclides for testing adsorption from simulated decontamination solutions. Both ^65^Zn^2+^ and ^60^Co^2+/3+^ are d-block metal cations with similar properties to other radioactive metal cations present in decontamination solutions, such as ^110m^Ag^+^, ^124^Sb^3+^, ^59^Fe^2+/3+^, and ^54^Mn^2+^.

As presented in Table 1, the distribution coefficients for ^60^Co and ^65^Zn are very high, even in the presence of competitive cations.

**Figure 3:**
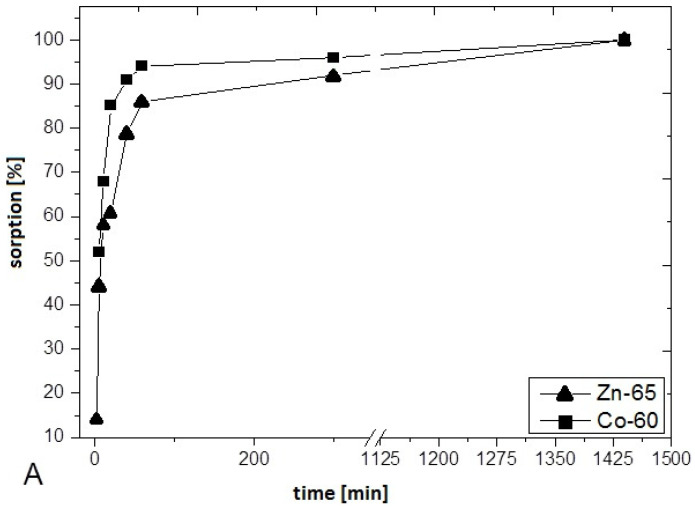
Sorption percentages of ^60^Co and ^65^Zn on TiO_2_ nanotubes as a function of time at pH = 4.

The kinetics of ^60^Co and ^65^Zn cation adsorptions onto TiO_2_ nanotubes were measured and analyzed. As shown in Figure 2, the adsorption processes of these two cations are relatively fast, and they are considerably faster than for ^137^Cs^+^ [10]. In the case of ^137^Cs, we previously found that ion exchange processes occur not only on the nanotube surfaces, but also on hydroxyl functional groups present inside the nanotubes [10]. In this case, the ion exchange must be preceded by desolvation of cations and their diffusion inside the tubes. Large, hydrated Co^2+^_(*aq*)_ and Zn^2+^_(*aq*)_ cations cannot intercalate in the very small channels of TiO_2_ nanotubes, so they are only adsorbed on surface functional groups, therefore allowing adsorption to occur much faster (Figure 3). 

**Figure 4:**
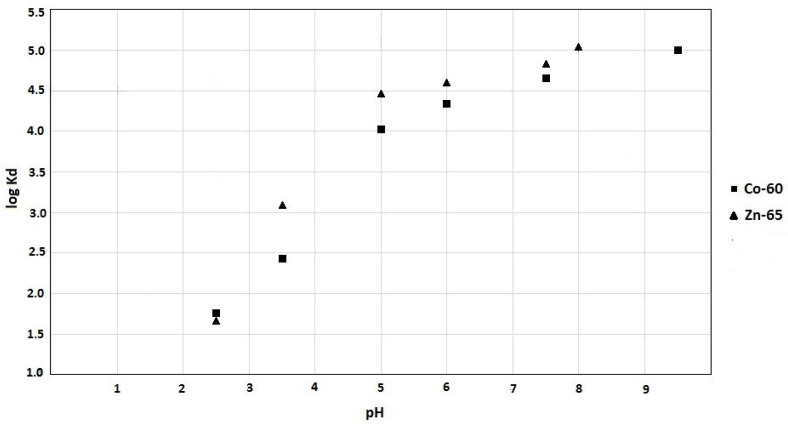
The dependence of ^60^Co and ^65^Zn distribution coefficients on solution pH.

Due to the weakly acidic character of the hydroxyl groups on TiO_2_ sorbents, the influence of pH on the adsorption process has been evaluated. As shown in Figure 4, the distribution coefficients for pH > 3 have values higher than 10^3^, which is sufficient for applying such TiO_2_ nanotubes for decontamination processes. 

### 3.2. Sorption of ^65^Zn and ^60^Co From Simulated Decontamination Fluids

We examined how the ligands found in decontamination fluids can affect the adsorption of ^65^Zn and ^60^Co on TiO_2_ nanotubes and amorphous titanium dioxide. We analyzed model solutions containing complexing ligands at specified concentrations (e.g., 10^−3^ M citric and oxalic acids, and 5 × 10^−4^ M EDTA) that are close to those found in common decontamination fluids.

**Figure 5:**
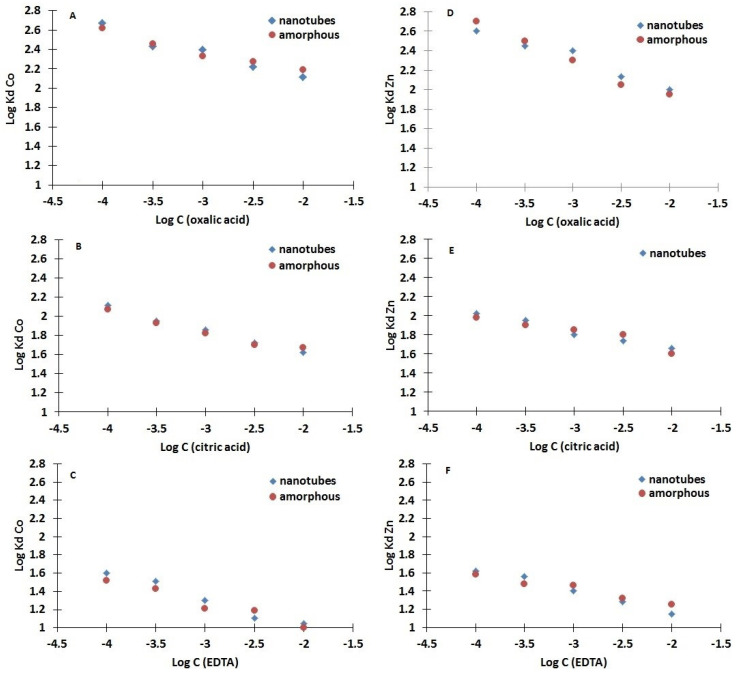
The dependence of ^60^Co (A-C) and ^65^Zn (D-F) cation distribution coefficients on the concentration of chelating agents

As shown in Figure 5, TiO_2_ nanotubes exhibit adsorption properties very similar to the amorphous hydrated TiO_2_. However, the presence of chelators significantly reduces sorption of ^65^Zn by both sorbents. This effect is observed at concentrations as low as 10^−4^ M. The most effective chelator is EDTA, which, even at a concentration of 10^−4^ M, decreases the adsorption of ^65^Zn to log *K*_d_ = 1.6. In contrast, the ^65^Zn log *K*_d_ was considerably higher (approx. 4.5) when measured for a solution without EDTA. Citric and oxalic acids do not form such strong complexes with Zn^2+^ ions, but nearly a 100-fold decrease in the distribution coefficient is still observed in the presence of these ligands at 10^−4^ M concentrations. At pH = 4, which is typical for decontamination fluids, but without the addition of any chelators, the log *K_d_* values are much higher and equal, respectively, 3.2 and 3.7 for Co-60 and Zn-65.

An analogous effect of ligand concentration on ^60^Co sorption was also observed and further investigated. Specifically, the ^60^Co-EDTA chelates appear to be the most thermodynamically stable, although there is also a significant decrease in *K*_d_ in the presence of increasing concentrations of the citric and oxalic acid ligands (Figure 5).

### 3.3. Photocatalytic Decomposition of Complexes in Simulated Decontamination Fluids 

The photochemical activity of TiO_2_ samples is related to their physicochemical properties including specific surface area, type and size of pores, degree of surface hydroxylation, degree of agglomeration of photocatalyst particles, degree of crystallization, and number of defects in the crystal structure [14]. Since the specific surface area of nanostructures is much larger than in µm-sized amorphous TiO_2_, the photoactivity of these nanostructures is relatively higher. In addition, the adsorption affinity of TiO_2_ for metal cations (e.g., Fe^2+^ and Cd^2+^) is generally size-dependent [15,16]. 

Experiments probing photocatalytic degradation followed by sorption were carried out for simulated decontamination fluids containing EDTA (5 × 10^−4^ M), oxalic acid (10^−3^ M), and citric acid (10^−3^ M), spiked with ^60^Co and ^65^Zn (Table 2). We tested TiO_2_ nanotubes and µm-sized amorphous TiO_2_, and compared these results with those obtained using commercial photocatalytic standard TiO_2_ P25 (rutile/anatase phase with an average primary particle size of 21 ± 5 nm). 

The obtained results clearly indicate that, among the tested TiO_2_ samples, only TiO_2_ nanotubes exhibit both high photocatalytic activity and sorption ability. After only 2 min of exposure to UV radiation, the decomposition of the complexes takes place, and due to the resulting liberation of Co^2+^ and Zn^2+^ ions, the *K*_d_ values for ^60^Co and ^65^Zn radionuclides rapidly increase. Before the photocatalytic degradation of chelators, the sorption of studied cations from simulated decontamination fluids on nanotubes was quite low, and log Kd was 1.4 for ^65^Zn and 1.3 for ^60^Co. After photocatalytic degradation, these values increased to 3.7 and 3.5, respectively. Low adsorption on amorphous TiO_2_ is caused by this material’s weak photocatalytic efficiency. Similar to amorphous TiO_2_, the commercial P25 nanoparticle sample displayed no increase in ^60^Co and ^65^Zn sorption.

Previous studies involving P25 showed that it is a very efficient photocatalyst, which easily decomposes EDTA, as well as citric and oxalic acids [17,18,19]. However, the calcination in the production process of P25 nanoparticles causes the release of interstitial water and the sorbent loses its ion exchange properties. 

The lower values of the distribution coefficients of both cations in the case of sorption on P25 after photocatalysis are also related to the smaller specific surface area of these nanoparticles compared to the obtained nanotubes. P25 nanoparticles have a specific surface area of about 50 m^2^/g, while the specific surface area of the synthesized nanotubes was 298 m^2^/g. [10]

The development of the specific surface of titanium dioxide influences the number of active sites. As Ao et al. found, the size of the active surface has a significant impact on the photo-oxidation process. The authors proved that the amount of hydroxyl radicals on the TiO_2_ surface increases with an increase in the specific surface area [20].

The results obtained for amorphous TiO_2_ and P25 samples are consistent with those from studies on the decomposition of complexes in simulated decontamination liquids by photolysis [13]. These photocatalytic degradation studies were carried out for a simulated decontamination fluid with a composition similar to that used in the present work (e.g., 0.01 M oxalate, 0.005 M citrate, and 0.005 M EDTA), and photocatalyst grains of various forms of titanium dioxide with diameters of about 1 μm were used (e.g., 100% rutile, 100% anatase, amorphous TiO_2(*aq*)_, and P25 particles (70% anatase, 30% rutile)). The authors observed photocatalytic degradation of the ligands and their complexes with Co^2+^ and Cr^3+^, with yields of 20–60% [13]. Among the tested catalysts, the best results were obtained using P25 particles, which demonstrate low sorption properties. Therefore, the decomposition of complexes was combined with sorption onto other effective inorganic ion exchangers, such as polyantimonic acid, zirconium phosphate, and zeolites [13].

Application of such nanotubes results in much simpler purification of decontamination fluids than in previous approaches.

## 4. Conclusions

This study demonstrates the efficiency and applicability of a one-step process for the separation of radionuclides from nuclear wastes containing complexing agents. A combination of photocatalytic degradation of metal-ligand complexes and sorption of the liberated radionuclides onto TiO_2_ nanotubes offers a promising route for the treatment of spent decontamination fluids. In order to apply this process, it is still necessary to develop technology for the effective separation of very small TiO_2_ nanotubes from decontamination solutions.

## Figures and Tables

**Table 1:** Comparison of log(*K*_d_) of the main radionuclides present in decontamination fluid solutions containing competitive Na^+^ and K^+^ cations at pH = 4.

Radionuclide (Trace Concentration)	Log K_d_ in the Presence of	
	0.1 M Solution NaNO_3_	0.1 M Solution KNO_3_
^137^Cs	2.6	2.5
^85^Sr	4.9	4.8
^65^Zn	3.3	3.4
^60^Co	4.3	3.7

**Table 2:** Adsorption of ^60^Co and ^65^Zn on TiO_2_ sorbents from simulated decontamination solutions (pH = 4) containing EDTA (5 × 10^−4^ M), oxalic acid (10^−3^ M), and citric acid (10^−3^ M), after photocatalytic decomposition of the ligands.

Type of Sorbent	Radionuclide	Log Kd after Photocatalysis
**nanotubes**	^65^Zn	**3.73 ± 0.08**
	^60^Co	**3.49 ± 0.04**
**P25**	^65^Zn	1.11 ± 0.07
	^60^Co	0.93 ± 0.08
**amorphous TiO_2_**	^65^Zn	1.01 ± 0.06
	^60^Co	0.96 ± 0.04
